# Household Firearm Storage Practices

**DOI:** 10.1001/jamanetworkopen.2025.18960

**Published:** 2025-07-03

**Authors:** Kelsey M. Conrick, Samantha Banks, Julia P. Schleimer, Anthony Gomez, Aley Joseph Pallickaparambil, Myduc Ta, Ali Rowhani-Rahbar

**Affiliations:** 1 Department of Epidemiology, School of Public Health, University of Washington, Seattle; 2 Firearm Injury & Policy Research Program, Department of Pediatrics, School of Medicine, University of Washington, Seattle; 3 Health Sciences Division, Public Health—Seattle & King County, Washington; 4 Department of Health Systems and Population Health, School of Public Health, University of Washington, Seattle

## Abstract

**Importance:**

Secure firearm storage is a key strategy for firearm injury prevention. Understanding trends in firearm storage behaviors over time is critical for developing tailored interventions.

**Objective:**

To characterize trends in household firearm storage from 2013 to 2022 in Washington State and to assess time-varying associations between demographic groups and secure storage.

**Design, Setting, and Participants:**

This survey study with repeated cross-sectional measures used data from the Behavioral Risk Factor Surveillance System, a state-based survey of adults (aged ≥18 years) living in Washington State that was conducted by telephone and included questions regarding firearm storage in the 2013, 2015, 2016, 2018, 2020, or 2022 cycles. Data were analyzed from August 2024 through April 2025.

**Exposures:**

Demographic variables including sex, age, rurality (urban vs small town or rural), veteran status, and the presence of children in the home.

**Main Outcomes and Measures:**

The primary outcome was the percentage of adults who reported secure firearm storage (all household firearms stored unloaded and locked). Logistic regression models that included each of the selected demographic variables, year, and interactions between them were used to estimate time-varying associations, with weighted percentages used to account for complex survey design and to be representative of the adult population of Washington State.

**Results:**

A total of 77 275 survey respondents (mean [SD] age, 46.7 [18.9] years) in Washington State were included in the analysis. Of these, 51.7% (95% CI, 51.2%-52.1%) were female. The percentage of adults reporting household firearm presence was 35.7% (95% CI, 34.4%-37.0%) in 2013 and 33.3% (95% CI, 32.5%-34.1%) in 2022. Among households with firearms (n = 27 721), secure storage was reported among 34.9% (95% CI, 32.8%-37.1%) in 2013 and 48.8% (95% CI, 47.2%-50.3%) in 2022. The odds of secure storage significantly increased per year overall (odds ratio [OR], 1.07; 95% CI, 1.06-1.08), and among nearly all subgroups: males (OR, 1.08; 95% CI, 1.07-1.10), females (OR, 1.06; 95% CI, 1.04-1.08), veterans (OR, 1.09; 95% CI, 1.07-1.10), nonveterans (OR, 1.07; 95% CI, 1.05-1.08), those with children (1.08; 95% CI, 1.06-1.10), and those without (OR, 1.07; 95% CI, 1.06-1.08). The odds increased in urban areas (OR, 1.07; 95% CI, 1.05-1.08) and suburban areas or large towns (OR, 1.08; 95% CI, 1.05-1.10) but not in small towns or rural areas (OR, 1.01; 95% CI, 0.97-1.06).

**Conclusions and Relevance:**

In this survey study of Washington State adults, the percentage of adults reporting secure storage of household firearms increased from 2013 to 2022, but differences persisted across demographic groups. These findings underscore the need for more tailored interventions to address these disparities.

## Key Points

**Question:**

How have overall and demographic group–specific trends in household firearm storage changed in Washington State from 2013 to 2022?

**Findings:**

In this survey study with repeated cross-sectional measures involving 77 275 adult respondents of whom 27 721 lived in firearm-owning households in Washington State, the proportion of adults reporting secure household firearm storage (locked and unloaded) increased from 34.9% in 2013 to 48.8% in 2022. These increases were observed among most key demographic groups (sex, age, location, veteran status, and presence of a child in the home), despite differences in the proportion of adults reporting secure firearm storage across these groups.

**Meaning:**

The findings suggest that although the proportion of adults reporting secure household firearm storage has increased from 2013 to 2022 in Washington State, differences across demographic groups underscore the need for tailored interventions to promote secure firearm storage.

## Teaser

This survey study uses data from the Behavioral Risk Factor Surveillance System to assess trends in adults reporting secured household firearm storage in Washington State from 2013 to 2022.

## Introduction

The presence of a firearm in the home has been reported to increase the risk of death by suicide,^1^ homicide,^2^ unintentional discharge,^3^ and intimate partner homicide^4^ for household members. Secure storage practices have been associated with reductions in suicide and unintentional firearm injuries and deaths among youth and may prevent theft.^5,6^

Firearm ownership and purchase often vary by demographic groups across time; for example, women represented 50% of first-time firearm purchasers during the COVID-19 pandemic, despite representing only 37% of firearm owners overall in the US.^7^ Firearm storage practices additionally vary based on other characteristics, such as sex, veteran status, reasons for ownership, and the presence of children in the home, among others.^8,9,10^ For example, households with children are more likely to lock firearms, while individuals who have firearms primarily for self-defense may be less likely to use locking devices.

Understanding how firearm storage practices change over time and across population subgroups can inform timely and tailored public health strategies to reduce the burden of firearm injuries and deaths. For example, during spikes in firearm purchasing, tailored outreach to new owners, particularly those who are already motivated to secure firearms securely (eg, households with children), could build motivation to store firearms securely. Similarly, persistent gaps in storage among specific groups can guide strategies to address group-specific barriers. While some research has examined firearm storage practices in single cross-sections of time,^8,9,10^ there is a dearth of studies examining statewide trends over time, likely stemming from a lack of repeated measures data. Washington State administers the Behavioral Risk Factor Surveillance System (BRFSS) annually, with state-added questions about firearm storage annually or biennially in selected years from 2013 to 2022 offering a unique opportunity to assess trends over time. Our study sought to evaluate whether household firearm storage practices in Washington State changed over time and whether these patterns differ across key demographic subgroups.

## Methods

This survey study with repeated cross-sectional measures followed the American Association for Public Opinion Research (AAPOR) reporting guideline for survey studies.^11^ In accordance with the Common Rule, this study was exempt from review and informed consent because it used publicly available deidentified data and did not constitute human participant research.

### Sample

With support from the Centers for Disease Control and Prevention (CDC), each state administers the BRFSS questionnaire to noninstitutionalized adults aged 18 years and older to collect information related to health-risk behaviors, chronic health conditions, and use of preventive services based on a dual-frame, random-digit–dialed landline and mobile phone design.^12^ This survey is conducted by telephone and takes about 25 minutes. In addition to collection of answers to a standardized core questionnaire, states may also choose to add optional modules or state-added questions. Washington State added questions on household presence of a firearm and storage in 2013, 2015, 2016, 2018, 2020, and 2022. During the study period, the combined landline and mobile phone response rate ranged from a low of 31.1% in 2013 to a high of 50.0% in 2018. Complex survey weights were used to adjust responses to ensure that they were representative of the state adult population each year.^13^ Additional details on eligibility, screening, and administration are available on the CDC website.^13^

### Measures

#### Presence and Storage of Firearms

The firearm safety module begins with this text: “The next questions are about safety and firearms. We are asking these in a health survey because of our interest in firearm-related injuries. Firearms include weapons such as pistols, shotguns, and rifles. In answering the questions, do not include BB guns, starter pistols, or guns that cannot fire. Include those kept in a garage, outdoor storage area, car, truck, or other motor vehicle.” Hereafter in this article, we use the term *home* to capture all these settings. The 4 firearm-related questions included: (1) “Are any firearms now kept in or around your home?”; (2) “Is there a firearm in or around your home that is now loaded?”; (3) “Is there a firearm in or around your home that is unlocked?”; and (4) “Are any of the loaded firearms also unlocked?” For all 4 questions, the answer options are, “yes,” “no,” “don’t know,” or “refused.” We created a dichotomous variable to describe household firearm storage according to the state’s statutory definition.^14^ Secure storage was defined as all household firearms locked and unloaded; unsecured storage (reference group) was defined as any household firearm loaded, unlocked, or loaded and unlocked. Participants who could not be categorized into a storage category due to missing, “don’t know,” “refused,” or contradictory answers were excluded (n = 887 [3.1%]).

#### Individual- and Household-Level Demographic Characteristics

Individual-level characteristics considered for the trend analyses included respondent sex, age group, and veteran status. Household-level characteristics included the presence of any children (aged <18 years) and rurality. BRFSS categorizes zip codes into 4-tier Rural Urban Commuting Area codes (urban, suburban, large town, and small town or rural)^15^; due to small sample sizes, we collapsed suburban and large town. Race and Hispanic ethnicity (self-reported) were used descriptively to characterize the sample but were not used for trend analyses due to small sample sizes in the storage variable for Asian, Black or African American, and Native Hawaiian or Pacific Islander respondents.

### Statistical Analysis

Descriptive statistics were used to characterize the presence of a firearm, firearm storage, and demographic characteristics. All percentages were weighted according to the CDC’s guidance for iterative proportional fitting with 95% CIs, and χ^2^ tests were used to assess for differences in firearm presence and storage by respondent and household characteristics.^13^ To estimate time-varying associations between demographic variables and secure firearm storage, we conducted a series of separate logistic regression models. Each model included the survey year recorded as the number of years from the start of the study period (2013 = 0, 2015 = 2, 2016 = 3, and so on), the demographic variable of interest, and an interaction term between the demographic variable and year. To aid interpretability, we used linear combinations of coefficients to estimate the odds ratio (OR) for change in secure storage over time within each level of the demographic. Global likelihood ratio tests were used to assess the significance of interaction terms (*P* < .05). A sensitivity analysis to examine missingness in responses to the firearm presence question was conducted using χ^2^ tests. All analyses accounted for the BRFSS complex survey design using the survey package in R, version 2022.07.2 (R Project for Statistical Computing). We specified survey weights, strata, and primary sampling units according to CDC documentation and applied these to both descriptive statistics and regression models. Logistic regression models were fit using R svyglm, version 4.4-2, which uses weighted likelihood estimation and produces SEs that account for the complex sample design. Data were analyzed from August 2024 through April 2025.

## Results

### Sample Composition

Throughout the study period (2013-2022), there were 77 275 respondents in Washington State (mean [SD] age, 46.7 [18.9] years), including 10.5% (95% CI, 10.2%-10.8%) who identified as Hispanic, 8.8% (95% CI, 8.5%-9.2%) as non-Hispanic Asian, 3.3% (95% CI, 3.1%-3.5%) as non-Hispanic Black, and 69.9% (95% CI, 69.4%-70.4%) as non-Hispanic White (Table 1). Of these respondents, 51.7% (95% CI, 51.2%-52.1%) were female, 48.3% (95% CI, 47.9%-48.8%) were male, and 11.5% (95% CI, 11.2%-11.7%) were veterans. Among respondents, 33.0% (95% CI, 32.5%-33.4%) reported the presence of a child in the home, and 14.1% (95% CI, 13.8%-14.5%) reported living in a small town or rural area.

**Table 1.** Characteristics of Respondents and Households by Presence of a Firearm in or Around the Home Aggregated From 2013 to 2022

Characteristic	Weighted, % (95% CI)		
	Overall	Firearm in or around the home	No firearm in or around the home
Age group, y			
18-29	20.1 (19.7-20.6)	17.1 (16.4-17.8)	21.6 (21.1-22.2)
30-44	26.0 (25.5-26.4)	23.5 (22.8-24.3)	27.2 (26.7-27.8)
45-59	23.6 (23.2-24.0)	25.4 (24.7-26.1)	22.7 (22.2-23.2)
≥60	30.3 (29.9-30.7)	33.9 (33.3-34.6)	28.4 (27.9-28.9)
Race and ethnicity			
American Indian or Alaska Native	1.4 (1.3-1.5)	1.4 (1.2-1.6)	1.4 (1.2-1.5)
Asian	8.8 (8.5-9.2)	3.9 (3.5-4.3)	11.4 (10.9-11.8)
Black	3.3 (3.1-3.5)	1.7 (1.4-1.9)	4.1 (3.8-4.4)
Hispanic	10.5 (10.2-10.8)	4.7 (4.3-5.1)	13.5 (13-13.9)
Native Hawaiian or Pacific Islander	0.6 (0.5-0.6)	0.4 (0.3-0.5)	0.6 (0.5-0.7)
White	69.9 (69.4-70.4)	82.4 (81.7-83.1)	63.5 (62.9-64.1)
Multiracial	3.2 (3-3.3)	3.2 (2.9-3.5)	3.1 (2.9-3.4)
Other race^a^	0.5 (0.5-0.6)	0.5 (0.4-0.5)	0.5 (0.5-0.6)
“Don’t know,” “refused,” or missing	1.8 (1.7-2.0)	1.8 (1.6-2.0)	1.9 (1.7-2.0)
Sex			
Female	51.7 (51.2-52.1)	43.8 (43.0-44.6)	55.7 (55.1-56.3)
Male	48.3 (47.9-48.8)	56.2 (55.4-57.0)	44.3 (43.7-44.9)
Rurality			
Urban	71.0 (70.6-71.4)	62.1 (61.4-62.8)	75.6 (75.1-76.1)
Suburban or large town	10.2 (9.9-10.5)	13.7 (13.1-14.2)	8.5 (8.1-8.8)
Small town or rural	14.1 (13.8-14.4)	20.9 (20.3-21.4)	10.6 (10.3-10.9)
Missing	4.7 (4.5-4.9)	3.4 (3.1-3.7)	5.4 (5.1-5.7)
Veteran status			
Nonveteran	88.4 (88.1-88.7)	81.6 (81-82.2)	91.9 (91.6-92.2)
Veteran	11.5 (11.2-11.7)	18.3 (17.7-18.9)	8.0 (7.7-8.3)
Missing	0.2 (0.1-0.2)	0.1 (0.1-0.2)	0.2 (0.1-0.2)
Children aged <18 y in home			
Children present	33.0 (32.5-33.4)	31.0 (30.3-31.8)	33.9 (33.3-34.5)
No children present	66.5 (66-67)	68.6 (67.8-69.4)	65.4 (64.8-66)
Missing	0.5 (0.5-0.6)	0.4 (0.3-0.5)	0.6 (0.5-0.7)

^a^ Not specified in the Behavioral Risk Factor Surveillance System.

### Presence of a Firearm in the Home

**Figure 1.**
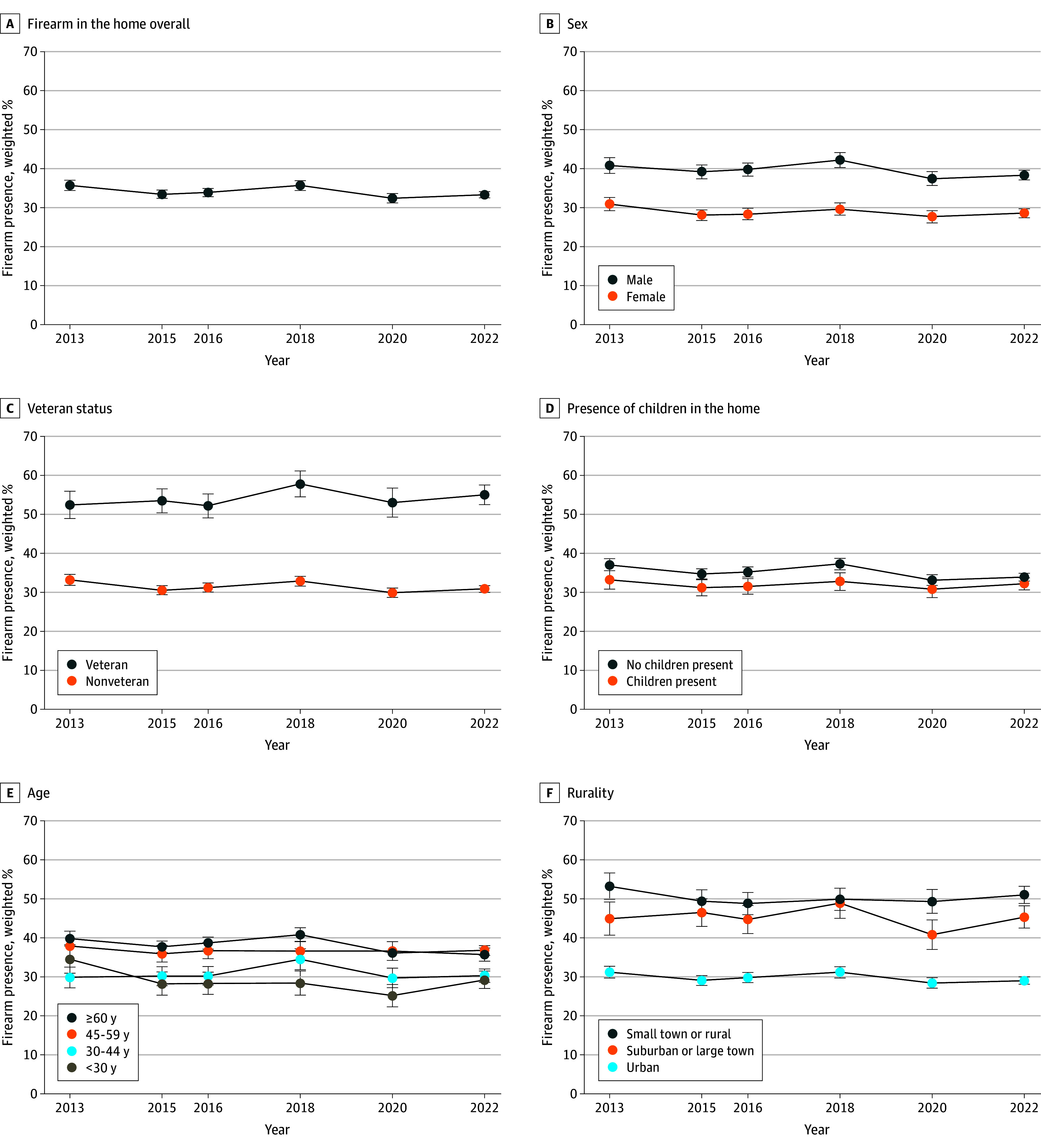
Trends in the Presence of a Firearm in or Around the Home by Characteristics of Respondents and Households in the Washington State Behavioral Risk Factor Surveillance System, 2013 to 2022 Error bars represent 95% CIs.

The percentage of respondents reporting the presence of a firearm in the home was 35.7% (95% CI, 34.4%-37.0%) in 2013 and 33.3% (95% CI, 32.5%-34.1%) in 2022 (Figure 1). Across the study period, there were differences between those reporting presence of a firearm in the home vs no firearm in terms of respondent age, race and ethnicity, sex, and veteran status, as well as household presence of a child and rurality (Table 1). For example, 56.2% (95% CI, 55.4%-57.0%) of male respondents reported the presence of a firearm in the home compared to 43.8% (95% CI, 43.0%-44.6%) of female respondents. Patterns in response missingness to the household presence of a firearm question were evident by age, sex, race and ethnicity, veteran status, rurality, and presence of a child in the home (eTable in Supplement 1).

### Firearm Storage

**Figure 2.**
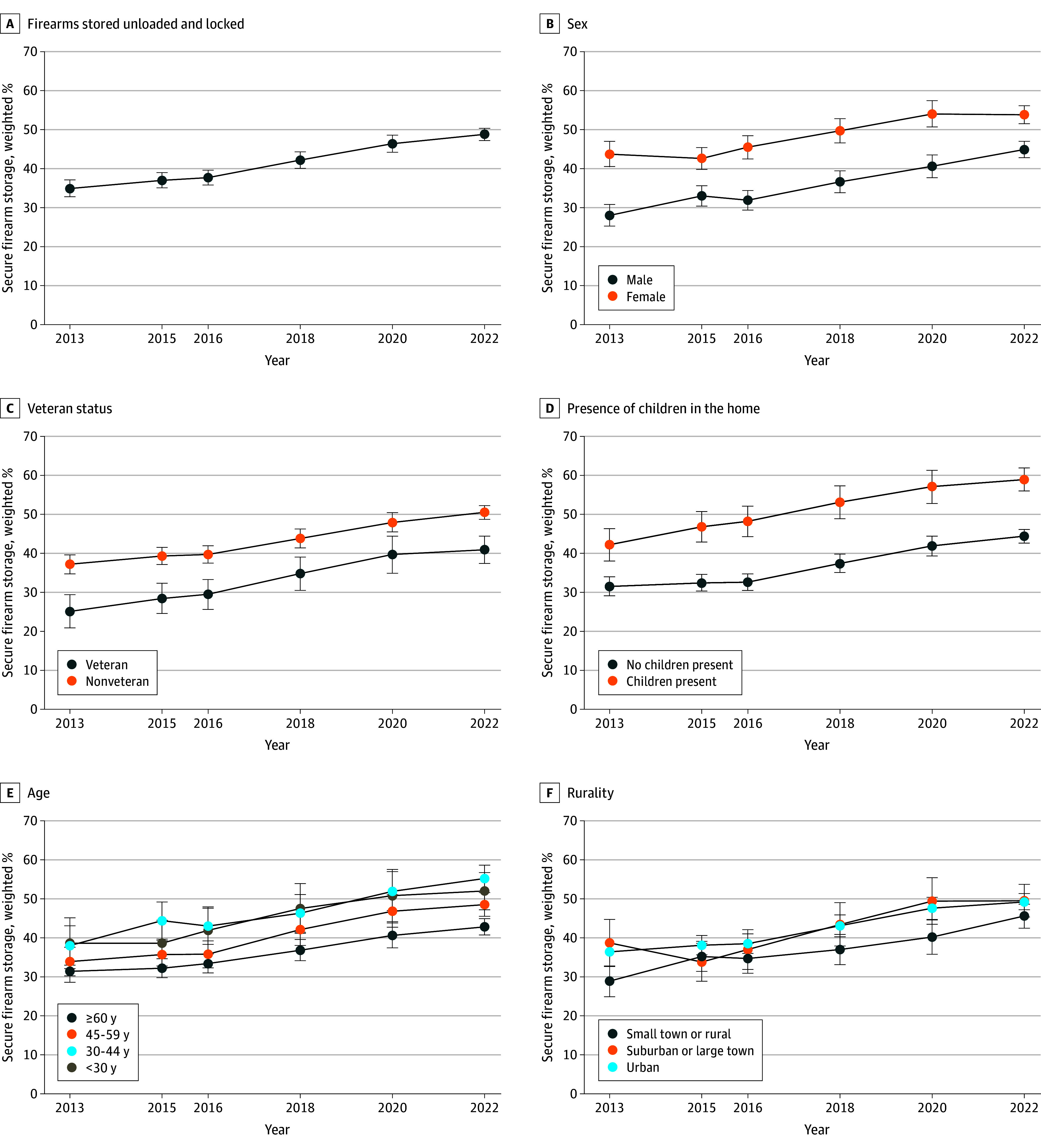
Trends in Secure Storage of Firearms in or Around the Home by Characteristics of Respondents and Households in the Washington State Behavioral Risk Factor Surveillance System, 2013 to 2022 Error bars represent 95% CIs.

Across the study period and among 27 721 respondents from households with firearms, 42.2% (95% CI, 41.1%-43.0%) indicated that firearms were stored securely (unloaded and locked); this percentage was 34.9% (95% CI, 32.8%-37.1%) in 2013 and 48.8% (95% CI, 47.2%-50.3%) in 2022 (Figure 2). On average across the study period, there were differences between those reporting secure (vs unsecured) household firearm storage in terms of respondent age, race and ethnicity, sex, and veteran status, as well as household presence of a child and rurality (Table 2). For example, 62.1% (95% CI, 61.1%-63.3%) of male respondents stored firearms unsecured compared to 37.9% (95% CI, 36.9%-38.9%) of female respondents.

**Table 2.** Characteristics of Respondents and Households by Household Firearm Storage Aggregated From 2013 to 2022

Characteristic	Weighted, % (95% CI)	
	Firearms stored secured (unloaded and locked)	Firearm stored unsecured^a^
Age group, y		
18-29	18.1 (17.0-19.3)	15.7 (14.8-16.6)
30-44	27.0 (25.8-28.1)	21.4 (20.4-22.3)
45-59	24.9 (23.8-25.9)	26.1 (25.2-27)
≥60	30.0 (29.0-31.0)	36.9 (36.0-37.8)
Race and ethnicity		
American Indian or Alaska Native	1.4 (1.1-1.7)	1.5 (1.2-1.7)
Asian	5.0 (4.3-5.8)	2.8 (2.3-3.3)
Black	1.8 (1.4-2.2)	1.6 (1.3-1.9)
Hispanic	4.9 (4.4-5.5)	4.5 (4-5)
Native Hawaiian or Pacific Islander	0.4 (0.2-0.6)	0.4 (0.3-0.6)
White	80.6 (79.5-81.8)	84.1 (83.2-84.9)
Multiracial	3.7 (3.1-4.3)	2.8 (2.5-3.2)
Other race^b^	0.4 (0.3-0.5)	0.5 (0.3-0.6)
“Don’t know,” “refused,” or missing	1.6 (1.3-1.9)	1.8 (1.5-2.0)
Sex		
Female	49.8 (48.5-51.1)	37.9 (36.9-38.9)
Male	50.2 (48.9-51.5)	62.1 (61.1-63.1)
Rurality		
Urban	63.4 (62.2-64.6)	61.2 (60.3-62.2)
Suburban or large town	13.8 (13-14.6)	13.6 (12.9-14.3)
Small town or rural	18.9 (18-19.8)	22.4 (21.6-23.1)
Missing	3.9 (3.4-4.4)	2.8 (2.4-3.2)
Veteran status		
Nonveteran	85.0 (84.1-85.8)	78.5 (77.7-79.3)
Veteran	14.9 (14.1-15.8)	21.4 (20.5-22.2)
Missing	0.1 (0-0.2)	0.1 (0.1-0.2)
Children aged <18 y in home		
Children present	38.2 (37-39.5)	25.8 (24.8-26.8)
No children present	61.4 (60.2-62.7)	73.8 (72.9-74.8)
Missing	0.3 (0.2-0.5)	0.4 (0.2-0.5)

^a^ Includes participants whose firearms were stored unlocked and loaded, loaded and locked, or unlocked and unloaded.

^b^ Not specified in the Behavioral Risk Factor Surveillance System.

Over the study period, the odds of firearms being stored securely increased annually on average (OR, 1.07; 95% CI, 1.06-1.08). The odds of secure storage significantly increased over time among nearly all subgroups: males (OR, 1.08; 95% CI, 1.07-1.10), females (OR, 1.06; 95% CI, 1.04-1.08), veterans (OR, 1.09; 95% CI, 1.07-1.10), nonveterans (OR, 1.07; 95% CI, 1.05-1.08), those with children (OR, 1.08; 95% CI, 1.06-1.10), and those without (OR, 1.07; 95% CI, 1.06-1.08). The odds increased in urban areas (OR, 1.07; 95% CI, 1.05-1.08) and suburban or large towns (OR, 1.08; 95% CI, 1.05-1.10), but not in small towns or rural areas (OR, 1.01; 95% CI, 0.97-1.06) (Table 3).

**Table 3.** Trends in Secure Storage of Firearms by Selected Demographic Characteristics Among Individuals Reporting a Firearm in or Around the Home in Washington State From 2013 to 2022 (27 721 Households)^a^

Selected characteristic	OR (95% CI)	*P* value for interaction^b^
Sex		
Female	1.06 (1.04-1.08)	.04
Male	1.08 (1.07-1.10)	
Veteran status		
Veteran	1.09 (1.07-1.12)	.12
Nonveteran	1.07 (1.05-1.08)	
Children aged <18 y present in the home		
Children present	1.08 (1.06-1.10)	.45
No children present	1.07 (1.06-1.08)	
Age group, y		
18-30	1.07 (1.04-1.11)	.73
30-44	1.08 (1.05-1.10)	
45-59	1.08 (1.06-1.10)	
≥60	1.06 (1.05-1.08)	
Rurality		
Urban	1.07 (1.05-1.08)	.83
Suburban or large town	1.08 (1.05-1.10)	
Small town or rural	1.01 (0.97-1.06)	

^a^ Race and Hispanic ethnicity were not used for trend analyses due to small sample sizes in the storage practices variable for Asian, Black or African American, Hispanic, Native Hawaiian or Pacific Islander, and respondents.

^b^ *P* for interaction comes from an interaction term between year (continuous) and each of the presented categories in the table.

In every year, the odds of secure household firearm storage were lower for veterans than nonveterans, although rates of change in these odds were not different over time. Likewise, the odds of secure firearm storage in the household were consistently lower for male respondents compared with female respondents, respondents in small towns or rural compared with urban areas, and respondents aged 60 years or older compared with respondents younger than 30 years (Table 3). The rates of change in these odds were also not different over time, except for sex, where the rate of change in household firearm storage for male respondents was higher than for female respondents (*P* for interaction = .04).

## Discussion

To our knowledge, this survey study is one of the first population-level statewide examinations of changes in household firearm storage over a decade, providing important insights into household firearm storage in Washington State from 2013 to 2022. We found that the percentage of adults reporting secure storage increased overall among households during the study period. This pattern was consistent across all groups of respondents and households examined. However, differences in firearm storage across these demographic groups by age, sex, and veteran status, as well as household-level factors, including the presence of a child in the home and rurality, persisted during the study period, indicating that targeted interventions may still be necessary to address the potential underlying factors contributing to differences in household firearm storage.

The public health implications of increases in secure storage are potentially substantial, as improved secure storage practices are associated with reduced risks of suicide and unintentional firearm injury, particularly among youth.^5,6^ Although household secure firearm storage has increased in Washington State over the past decade, half of all firearm-owing households still store at least one firearm unlocked or loaded. Prior cross-sectional studies have also found low proportions of secure firearm storage,^8,9,10,16,17,18,19^ although our estimates of secure storage are slightly higher than other samples. The increase in household secure storage observed in this study may reflect broader shifts in public attitudes and practices related to firearm safety. Washington State has taken multiple steps over the past decade to support secure firearm storage, including a 2019 law (Wash Rev Code §9.41.360) allowing criminal prosecution if an unsecured firearm is accessed by a prohibited person and is used to cause harm.^20^ In addition, state agencies and community organizations have implemented secure storage campaigns, educational initiatives, and public awareness efforts.^21^ Although our study was not designed to evaluate the impact of these efforts, future research could explore whether such policies and programs have contributed to changes in storage behavior.

Several interventions have been found to increase secure firearm storage, including in clinical^22^ and community settings.^23^ While locking device preferences differ based on motivations for ownership and types of firearms owned,^9^ most firearm owners prefer gun safes and lockboxes to other devices, such as trigger or cable locks.^9,24^ Furthermore, research suggests that it is potentially important for secure storage interventions to directly provide devices to individuals.^25^ Public health campaigns that highlight the risks of unsecured firearms, offer practical solutions, and provide tailored messaging may be effective in promoting secure storage; however, more research on this topic is needed. Results of our study suggest a need to further tailor secure firearm storage interventions and messages to specific populations, considering their unique experiences and contexts. While our study focused on trends in secure storage among firearm-owning households, future research could examine whether shifts in the demographic composition of these households over time play a role in observed changes in storage practices. Additionally, future research should consider differing definitions of secure storage (eg, locked but loaded) and consider how the phrasing of questions or data sources (eg, self-report vs observational methods) may influence findings.^26^

Our study found differences in household firearm storage by household rurality and the presence of a child in the home. Households in rural areas were more likely to have firearms stored unsecured, potentially due to cultural attitudes toward firearms, hunting traditions, and perceived need to access firearms quickly for protection from animals or people.^27^ Additionally, households with children were more likely to report firearms stored securely compared with those without children. This finding may reflect increased awareness of the risks that unsecured firearms pose to children, as well as the impact of targeted public health messaging and interventions aimed at parents and guardians to promote secure storage practices in homes with minors.^21,28^ However, differences in household secure storage remain, particularly in rural compared with urban areas. To address these gaps, interventions to promote secure firearm storage should be tailored. In rural areas, interventions involving local community leaders as trusted messengers, using rural health services to disseminate secure storage information, referring to locally derived data, and reflecting community values around responsible firearm ownership have been shown to be promising.^27,29,30^ Additionally, emphasizing the importance of secure firearm storage for households with children may be a key prevention strategy. In one study of firearm owners, preventing children from unauthorized access to firearms was the most common reason for considering locking unsecured firearms.^9^

The differences observed in household firearm storage by respondent sex, age, and veteran status could reflect true differences in household storage or differences in knowledge of or reporting about household storage, since the respondent may not be the owner of household firearms or responsible for storage decisions. We highlight these variables because they are individual-level characteristics of the respondent and thus may affect the validity of analyses regarding household firearm storage. For example, when women were the respondents, they reported firearms being stored more securely than when men were the respondents (ie, the person who answered the telephone was not necessarily representative of who owned the firearm and/or was responsible for storage decisions). However, since these differences are observed across different households, not within the same household, we cannot assess whether women and men living in the same household would report firearm storage differently. Similarly, differences in household firearm storage by respondent age and veteran status should be interpreted with caution, as these too may reflect differences in reporting rather than differences in behavior. Furthermore, shifts in ownership patterns may be affecting firearm storage practices, as new firearm owners may differ in their awareness of or adherence to secure storage guidelines.

### Limitations

Limitations of this study include potential recall and social desirability biases in self-reported data on firearm presence and storage. Differences in missing responses to the firearm presence question by age, sex, veteran status, rurality, and the presence of a child in the home suggest a possible selection bias. These variations in response patterns, particularly in 2020, may affect the analysis of storage trends during that year. Additionally, while some practices recommend storing firearms unloaded, locked, and with ammunition locked separately, the Washington State–added storage questions do not ask about ammunition storage, which may limit the comprehensiveness of our findings on secure storage behaviors.^31,32^ Similarly, the questions on household firearm presence do not specify whether the firearm is located in the home, car, shed, or other areas. Given differing rates of firearm theft across these locations,^33^ this distinction could have important implications for firearm storage practices. The questions also do not ask about the type of household firearm (eg, long gun and/or handgun); this limitation may be particularly important when interpreting the findings by rurality. Finally, our findings may not be generalizable outside of Washington State.

## Conclusions

This survey study with repeated cross-sectional measures contributes to the literature by examining trends and demographic differences in household firearm storage in Washington State from 2013 to 2022. While overall secure storage practices increased over the study period, differences remained across different demographic groups. Tailored messaging that considers the specific motivations and barriers of different groups of people living in firearm-owning homes coupled with practical solutions, such as providing free locking devices, may enhance the effectiveness of these initiatives. Future research should explore the trends and implications of more recent policy and societal changes, such as the surge in firearm purchases during the COVID-19 pandemic and changing demographics of firearm ownership, for storage behaviors to further inform public health prevention strategies.

## Notes

Supplement 1. **eTable.** Characteristics of respondents by response status of the question asking if a firearm is in or around the home (aggregated across study time period: 2013-2022)- Washington State Behavioral Risk Factor Surveillance System

Supplement 2.
Data Sharing Statement
