# Supplementary material for: Household Firearm Storage Practices
Source: JAMA Netw Open. 2025 Jul 3;8(7):e2518960. doi: 10.1001/jamanetworkopen.2025.18960 (PMC12232188; doi:10.1001/jamanetworkopen.2025.18960)
Supplement: Supplement 1. — eTable. Characteristics of respondents by response status of the question asking if a firearm is in or around the home (aggregated across study time period: 2013-2022)- Washington State Behavioral Risk Factor Surveillance System [file jamanetwopen-e2518960-s001.pdf]

Supplemental Online Content

Conrick KM, Banks S, Schleimer JP, Gomez A, Pallickaparambil AJ, Ta M, Rowhani-Rahbar A. Household firearm storage practices in Washington State, 2013 to 2022. *JAMA Netw Open.* 2025;8(7): e2518960 doi:10.1001/jamanetworkopen.2025.18960

eTable. Characteristics of respondents by response status of the question asking if a firearm is in or around the home (aggregated across study time period: 2013-2022)- Washington State Behavioral Risk Factor Surveillance System

This supplemental material has been provided by the authors to give readers additional information about their work.

© 2025 Conrick, KM et al. *JAMA Network Open*.

Table. Characteristics of respondents by response status of the question asking if a firearm is in or around the home (aggregated across study time period: 2013-2022)- Washington State Behavioral Risk Factor Surveillance System

	Response Status for Presence of Firearm In/Around Home		
	**Missing (n=16,436)** Unweighted n, %	**Not Missing (n=77,275)** Unweighted n, %	**P**
**Age Group (years)**			
18-24	1154 (7.0%)	3911 (5.1%)	<0.001
25-34	2432 (14.8%)	7273 (9.4%)	
35-44	2417 (14.7%)	9344 (12.1%)	
45-54	2618 (15.9%)	11477 (14.9%)	
55-64	2966 (18.0%)	16062 (20.8%)	
65+	4849 (29.5%)	29208 (37.8%)	
**Sex**			
Female	7820 (47.6%)	43035 (55.7%)	<0.001
Male	8616 (52.4%)	34240 (44.3%)	
**Rurality**			
Urban	8859 (53.9%)	48331 (62.5%)	<0.001
Suburban/Large Town	1564 (9.5%)	7883 (10.2%)	
Small Town/Rural	3651 (22.2%)	18092 (23.4%)	
Missing	2362 (14.4%)	2969 (3.8%)	
**Veteran Status**			
Non-Veteran	13299 (80.9%)	66483 (86.0%)	<0.001
Veteran	2737 (16.7%)	10670 (13.8%)	
Missing	400 (2.4%)	122 (0.2%)	
**Children (<18) in home**			
Children present	4462 (27.1%)	18192 (23.5%)	<0.001
No children present	10889 (66.3%)	58758 (76.0%)	
Missing	1085 (6.6%)	325 (0.4%)	

© 2025 Conrick, KM et al. *JAMA Network Open*.
