# Supplementary material for: Household Firearm Storage Practices
Source: JAMA Netw Open. 2025 Jul 3;8(7):e2518960. doi: 10.1001/jamanetworkopen.2025.18960 (PMC12232188; doi:10.1001/jamanetworkopen.2025.18960)
Supplement: Supplement 2. — Data Sharing Statement [file jamanetwopen-e2518960-s002.pdf]

Data Sharing Statement

Conrick. Household Firearm Storage Practices. *JAMA Netw Open*. Published July 03, 2025. doi:10.1001/jamanetworkopen.2025.18960

Data
Data available: No

Additional Information
Explanation for why data not available: Data are publicly available from the Washington State BRFSS.
